# A Proposed Classification and Treatment Algorithm for Rectus Diastasis: A Prospective Study

**DOI:** 10.1007/s00266-021-02739-w

**Published:** 2022-01-18

**Authors:** Evangelos Keramidas, Stavroula Rodopoulou, Maria-Ioanna Gavala

**Affiliations:** Kosmesis Aesthetic Plastic Surgery Center, Central Clinic of Athens, Ethnikis Antistaseos 9-11, Chalandri, 15232 Athens, Greece

**Keywords:** Rectus diastasis, Body-contouring, Follow-up studies, Body mass index, Abdominoplasty, Sutures

## Abstract

**Background:**

This study presents a classification system and treatment method to correct Rectus diastasis (RD) during abdominoplasty.

**Materials and methods:**

One hundred and sixty seven patients undergoing abdominoplasty were enrolled between April 2014 and January 2018. Forty-three patients did not present with RD and were excluded from the analysis. Mean age was 40.32 years, mean BMI was 23.84, and minimum follow-up was 24 months. A four-type (A: mild 2–3cm, B: moderate 3–5cm, C: severe 5–7cm, and D: very severe 7–9cm) classification system is described. A different treatment method is performed in each category using continuous and interrupted absorbable sutures. Postoperatively patients filled up a questionnaire that involved the level of pain, the postoperative day they performed specific indoor/outdoor activities, and the evaluation of the aesthetic result.

**Results:**

No statistically significant differences were observed between the four RD types regarding pain, complications, and return to specific activities. All types of RD had the same low rate complication profile. The seroma rate was 0.81%. The infection rate was 0.81%, and the thromboembolism and the pneumonic embolism rate was 0%. After 2–6 years of follow-up no clinical recurrence of rectus diastasis was observed. All reoperations (14.52%) were performed due to scar deformities. Mean pain score levels were very low (<1.5) and within a week most patients returned to specific indoor and outdoor activities. Most patients were extremely satisfied with the results.

**Conclusions:**

In this article, we present an updated classification system and treatment protocol to provide surgeons a safe and standardized method that produces high-quality aesthetic results.

**Level of evidence IV:**

This journal requires that authors assign a level of evidence to each article. For a full description of these Evidence-Based Medicine ratings, please refer to the Table of Contents or the online Instructions to Authors www.springer.com/00266.

**Supplementary Information:**

The online version contains supplementary material available at 10.1007/s00266-021-02739-w.

## Introduction

Rectus diastasis (RD) is an anatomical term describing a condition in which an abnormal distance along their length separates the two rectus muscles [1, 2]. RD manifests as a protruding midline due to the gradual thinning and widening of the linea alba, often combined with ventral abdominal wall laxity [3]. Controversy exists regarding what constitutes a normal inter-rectus distance and thus when it can be considered abnormal [1]. A separation of >2 cm is considered to be an RD [3].

The common goal of the numerous techniques for RD correction found in the literature is to restore the aesthetics and function of the abdominal wall. However, in conjunction with the published literature on surgical treatment of RD that is of low scientific and methodological quality, the wide range of presentation poses great challenges to consistency in its aesthetic and functional correction [4–6].

Regarding nomenclature and classification, these are important in the preoperative identification of the presence and severity of each element of the deformity and assist in achieving more consistent results. Several classification systems exist to define the spectrum of RD [3, 7–9].

This prospective study proposes an RD classification, treatment algorithm, and evaluation protocol that plastic surgeons could easily adapt. Our goal is to provide a complete yet viable and not time-consuming methodology for this specific procedure. This protocol can be tailored individually to all patients so that all plastic surgeons can share their "high scientific quality" results with the rest of the aesthetic society.

## Materials and Methods

Between April 2014 and January 2018, 189 patients aged between 18 and 68 years that underwent abdominoplasty were enrolled in the study. All patients were treated by the senior author. Only patients with a minimum of 24 months of follow-up were included. All patients signed a written informed consent before inclusion.

A datasheet with the following info was created:

- Patient characteristics: BMI, age, sex, previous surgeries (gynecological or in the abdomen), smoking, birth characteristics (number of children, twins, triplets, type of delivery)
- Rectus diastasis characteristics: width, position, concomitant hernias, techniques used to correct the diastasis and hernias.
- Surgery characteristics: simultaneous operations (liposuction, breast augmentation/reduction, etc.), surgical time, hospitalization.
- Complications: infection, hematoma, seroma, deep vein thrombosis, pulmonary embolism, scar hypertrophy/ hyperchrosis, keloid, dog-ear, and revision surgery.
- Patient's quality of life characteristics: All patients undergoing rectus diastasis correction completed a postoperative questionnaire. Pain is assessed on a scale from 1 to 10 (0=no pain, 1–3=minor pain, 4–6=moderate pain, 7–10=severe pain) the first five postoperative days. Day of return to specific indoor activities (cooking, washing dishes, climbing stairs), outdoor activities (driving, shopping up to 10kgs), and gymnastics were also noted. Patients that returned to these activities and did not mention any work impairment were considered fully functional. The evaluation of the aesthetic result using a GAIS 5-point scale (exceptional or very improved, improved, no result, worse) was performed at least 12-month post-surgery. Patients were advised to visit our clinic at 5days, 15days, 1month, 6months, and then yearly. If a patient could not visit our clinic or any data were missing, we contacted them via telephone to fill out the questionnaire/datasheet.
  - Complications, pain, and return to specific activities were compared between the different types of RD.

### Diagnosis

- The finger-width method [10, 11] is used preoperatively and postoperatively. It primarily functions as a screening tool to detect the presence or absence of RD.
- Ultrasound imaging (USI) is performed in all patients preoperatively to localize any concomitant hernia.
- During surgery, measurement of rectus diastasis is performed using a standardized ruler to ensure symmetric plication. The diastasis is better visualized if needed using electrocautery by stimulating the rectus muscles [12]. The intraoperative measurements are taken in maximum inspiration and are not significantly increased due to muscle relaxants given during the procedure [13, 14].

### Classification

**Fig. 1:**
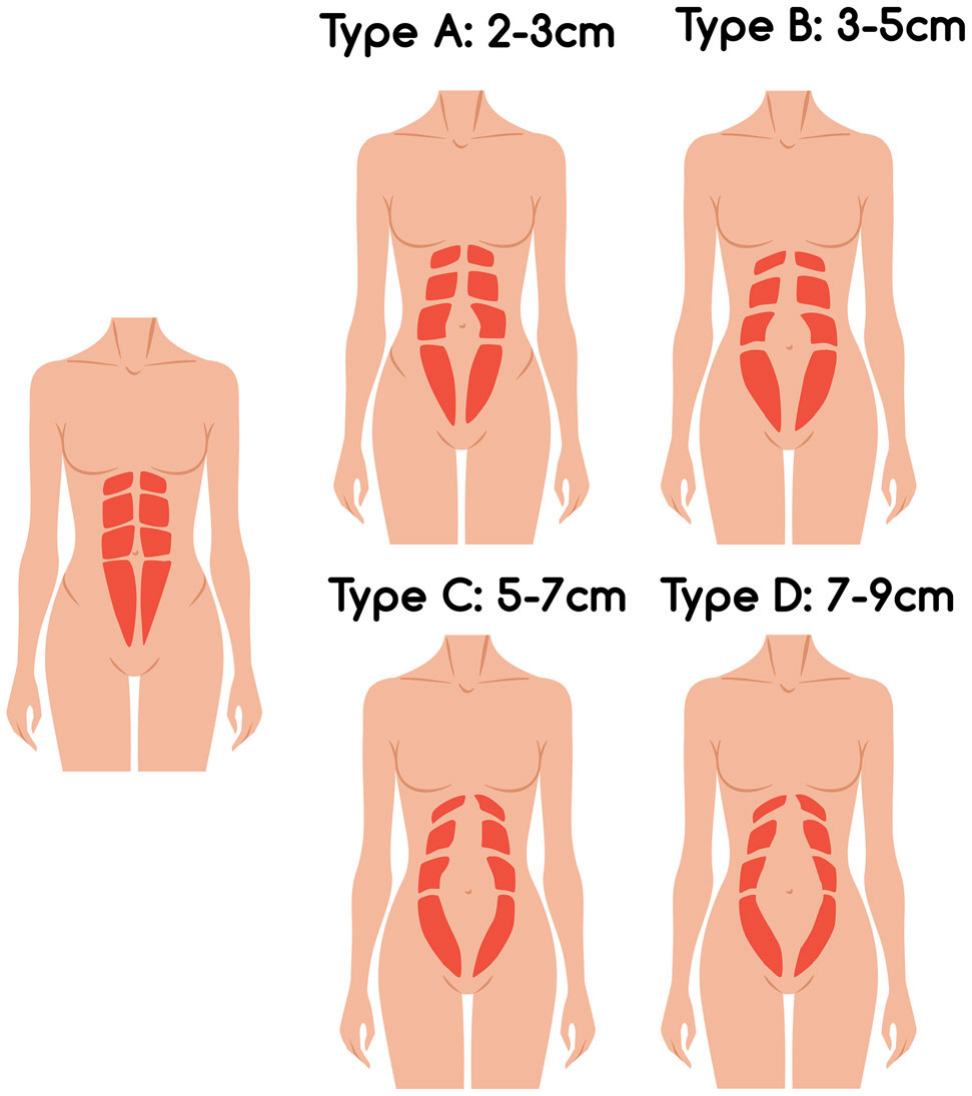
Schematic demonstration of rectus diastasis classification

A four-type classification system is described to define the deformities and tailor a treatment plan to each individual. Details are demonstrated in Table 1. An example of the RD classification is shown in Fig. 1.

**Table 1:** Four-type classification system and treatment algorithm

Type of rectus diastasis	Description of RD	Width of RD in cm	Surgical plan
A	Mild	2–3	CIS × 2
B	Moderate	3–5	CIS × 2 and interrupted sutures (2cm distance)
C	Severe	5–7	CIS × 2 and interrupted sutures (1cm distance)
D	Very severe	7–9	Ouroboros suture and interrupted sutures (2cm distance)

Only relevant aspects of the abdominoplasty procedure are mentioned.

### Preoperative Steps

A standardized antimicrobial and antithrombotic protocol are applied in every patient undergoing abdominoplasty.

The Caprini score (2005) [15] is calculated. Antithrombotic socks and Flowtron boots are placed before the surgery, and knees are bent 10–15° using a pillow. An electric blanket is used, surgical room temperature is set at 20–22°C and warm fluids are used to avoid hypothermia. Also, patients are advised to eliminate smoking and food supplements/aspirin/anti-inflammatory drugs two weeks before surgery. If indicated, chemoprophylaxis is used.

### Surgical Procedure

- All patients are submitted to the same procedure. Initially, we inject Klein solution in the flanks and the midline between the xiphoid and the umbilicus. Liposuction is performed to the flanks in most patients to define the waist better. The abdominoplasty starts with a horizontal suprapubic incision, and the dissection reaches the level of the umbilicus. Then we perform radiofrequency-assisted liposuction 3cm bilateral to the midline (epigastric area). Depending on the midline's thickness, using the pinch test, if it is more than 3cm, we perform liposuction 3cm bilateral to the midline (Greek Line technique) using a No3 cannula [16] If the thickness is less than 3cm, we do not perform liposuction—undermining proceeds above the abdominal fascia in a superior direction toward the xiphoid. No dissection is performed at the costal margins. The epigastrium area is dissected as a 6–8-cm-wide tunnel up to the xiphoid. During dissection, we leave a slight amount of fat on the fascia to increase the absorption surface and decrease the risk of seroma formation. After adequate dissection is completed, the correction or the recti muscles diastasis follows. The diastasis is measured subxiphoidally, epigastrically, umbilically, infraumbilically, and suprapubically. Categorization is based on the position of the maximum diastasis. The medial edges of the rectus fascia are plicated using absorbable sutures. (Ethicon polydioxanone II, size 0 sutures, Johnson &Johnson, New Brunswick, N.J.). Regardless of the maximum diastasis position (umbilical, epigastric, etc.), sutures are placed all along the xyphoid–pubis distance to strengthen the abdominal wall and improve the abdominal contour and waistline.
  The following techniques are used for RD correction:
- In Type A (2–3cm), a Continuous interlocking suture (CIS) is placed from the umbilicus to the xiphoid and another from the umbilicus to the pubis. This maneuver creates one suture layer. (CIS × 2)
- In Type B (3–5cm), a CIS × 2 suture is placed. Then interrupted sutures with a 2cm distance between them are placed from the pubis to the xiphoid—this maneuvers two suture layers.
- In Type C (5–7cm), a CIS × 2 suture is placed, and then interrupted sutures are placed with a 1cm distance between them. (two suture layers)
- In Type D (7–9cm), a traditional interlocking suture is performed from the umbilicus to the xiphoid and from the umbilicus to the pubis. Starting from the umbilicus when the xiphoid has been reached, the suture is knotted and continuous in the opposite direction: from the xiphoid to the umbilicus. This maneuver creates a stronger layer of continuous suture for maximum support of the rectus fascia in severe diastasis. Another same suture is placed from the umbilicus to the pubis. We named these sutures "ouroboros sutures" to describe better the surgical movement that resembles a snake eating its tail. The term derives from Ancient Greek οuροβoρος, from 'ouρα- 'tail' plus '-βορoς -boros '-eating.' Then interrupted sutures with a 2cm distance between them are placed from the pubis up to the xiphoid. This maneuver creates three suture layers. (See Video 1, which demonstrate the operative technique for correction of each type of rectus diastasis)

If hernia coexists, we correct it in conjunction with the rectus sheath plication using either sutures or mesh.

After plication, 20mg of ropivacaine 7.5mg are injected into the muscle using a 26-gauge needle.

After RD is corrected, we proceed with the excision of excess skin. Placement of high tension sutures to the central abdominal flap follows to pull the flap downwards. Two Penrose drains are placed (for up to 20 hours), and the abdomen is closed in a multilayer fashion using absorbable sutures.

### Postoperative Treatment

Flowtron boots and electric blanket/room temperature are used until the patient is discharged from the hospital. Patients are mobilized in 4–6 hours (10minutes walk every hour) after the operation to prevent thromboembolism. All patients are discharged in less than 24 hours. We advise patients to wear antithrombotic stockings for 15 days postoperatively. In Types A and B, if liposuction is performed, the corset was not used the first two weeks because we have noticed it increases the pain level. Patients wear the corset two weeks after surgery for one month. This way, the pain level is low, and patients are more comfortable. If liposuction is performed in Type C & D, a corset is also worn two weeks after surgery for one month. Reasons are to avoid pain caused by the corset and to avoid a further increase of intra-abdominal pressure. Patients are advised to return in less than 24hours to their everyday indoor activities (first, they can wash dishes, then cook, and when they feel comfortable to climb stairs) and avoid heavy labor (> 10kgs) for one month postoperatively. If they feel any discomfort, we recommend they use paracetamol. No morphine or stronger analgesic treatment is used neither perioperatively nor postoperatively.

### Statistical Analysis

All statistical analysis was carried out with MATLAB. Pairwise correlation coefficients were performed between measurements of rectus diastasis, comparing demographic data, surgery characteristics, complications, return to activities, and reoperations with the width measured at the surgery. ANOVA and Kruskal–Wallis were used to test for differences in several factors (e.g., BMI, pregnancy, etc.) between the four RD types. All tests were considered statistically significant at a level of *p* < 0.05.

## Results

Table 2 summarizes the preoperative demographic characteristics of the 124 patients that were included in the statistical analysis. Twenty-two patients were excluded from the study because they live abroad and could not meet the criteria of the minimum required follow-up, and the rest 43 patients because they did not present with RD. Thus no RD correction was performed.

**Table 2:** Demographic characteristics

No of patients	124
Sex (Female/Male)	120/4
Age (Mean, Range)	40.32(18–68)
BMI (Mean, Range)	23.84(17–35)
Smokers	55
History of previous surgeries (abdomen, gynecological)	90
At least one pregnancy	98
Twin pregnancy	6
No of births per woman (Mean, Range)	2(1–5)
Cesarean	1.84(1–4)
Normal	1.85(1–3)

Table 3 summarizes the rectus diastasis and surgery characteristics of the 124 patients included in the study. Simultaneous operations performed in this population were the following: breast augmentation, breast mammoplasty /reduction, liposuction in other areas (excluding flanks and Greek line), gynecomastia, thigh lift, and blepharoplasty. The widest RD were observed 5cm above and 5cm below the umbilicus. Specifically, two patients had subxiphoid RD (mean 5.5cm), two had epigastric (mean size 3.25cm), 118 had umbilical (mean size 5.04cm), and two had hypogastric (mean 3.5cm).

**Table 3:** Rectus diastasis and surgery characteristics

No of patients With/Without RD	124/43
No of patients based on classification:	
Type A	8
Type B	47
Type C	43
Type D	26
No of patients with concomitant hernias (all under 2cm)	13
Epigastric	3
Umbilical	10
No of patients with simultaneous operations	53
Surgical time (mean, range)	3.9hrs(2–6)
Hospitalization time (mean, range)	20hrs (6–24)

All types of RD patients had the same low rate complication profile. Hematomas or skin necrosis were not observed. Seroma rate was 0.81% (1 patient), the infection rate was 0.81% (1 patient), and thromboembolism and pneumonic embolism rate was 0%. Scar complications rate (hypertrophy, keloid, hyperchrosis, dog-ear) was 17.74% (22 patients), and revision rate was 14.52% (18 patients), all due to scar complications. During the study's follow-up, no recurrence of RD or hernia was confirmed clinically using the finger-width method. No statistically significant (*p* > 0.05) differences were observed between the four RD types regarding complications, pain, and return to specific indoor/outdoor activities.

- Pain score (0–10):
  First post-op day: 1.29
  Second post-op day: 1.31
  Third post-op day: 1.13
  Fourth post-op day: 0.89
  Fifth post-op day: 0.77

**Fig. 2:**
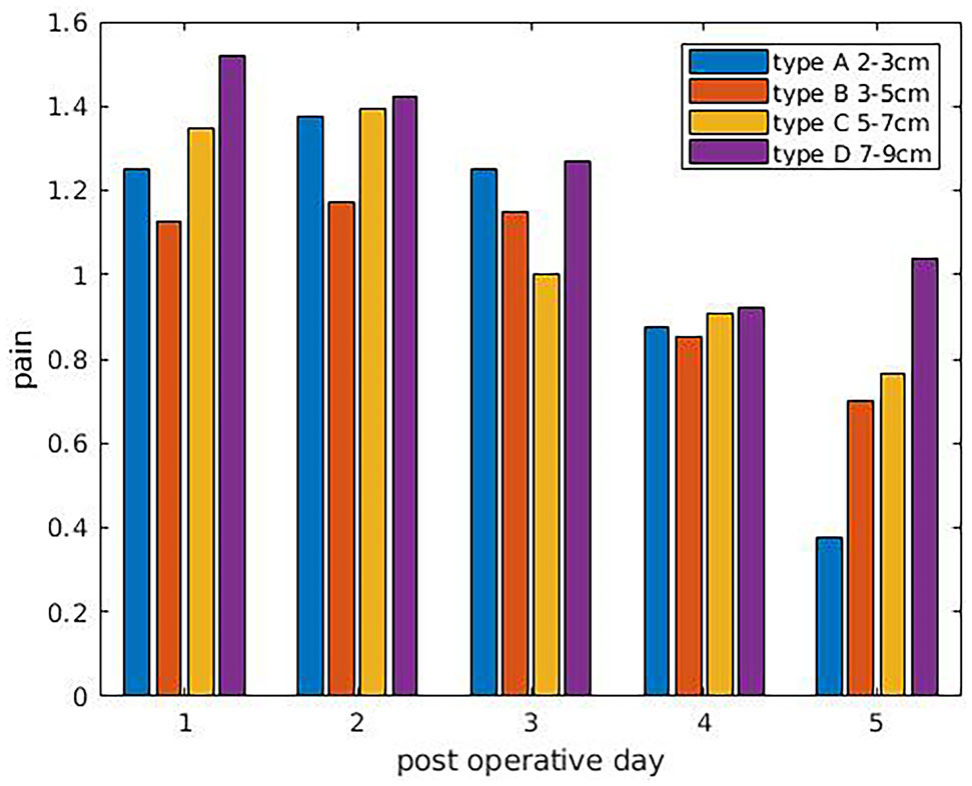
Mean values of pain for each RD category

No statistically significant (*p* > 0.05) differences were observed between the four RD types regarding pain. Mean values of pain for each RD category are shown in Fig. 2.

- Return to specific indoor activities:
  -cooking: 2.48 days
  -washing dishes: 1.39 days
  -climbing stairs: 2.90 day
- Return to specific outdoor activities:
  -driving: 4.91 days
  -shopping up to 10kgs: 6.10 days
  Gymnastics (at least two workouts/week): 4.33 months. Forty-two patients out of 124 started a training workout program for the first time after abdominoplasty

**Fig. 3:**
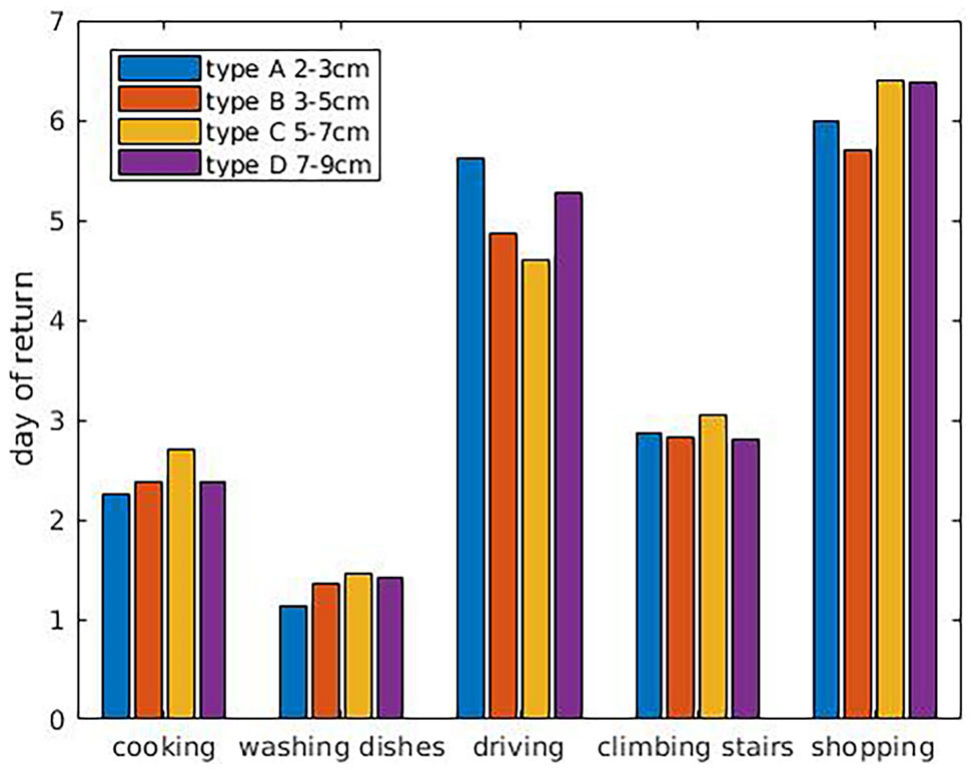
Mean values of return to specific activities for each RD category

Mean values of return to specific activities for each RD category are shown in Fig. 3. No statistically significant (*p* > 0.05) differences were observed between the four RD types regarding complications. Patients were very satisfied with the results. 95.97% marked the results as very much improved, 3.22% marked the results as much improved, 0.81% marked the results as improved, and 0% as no change/worse.

### Case 1

**Fig. 4:**
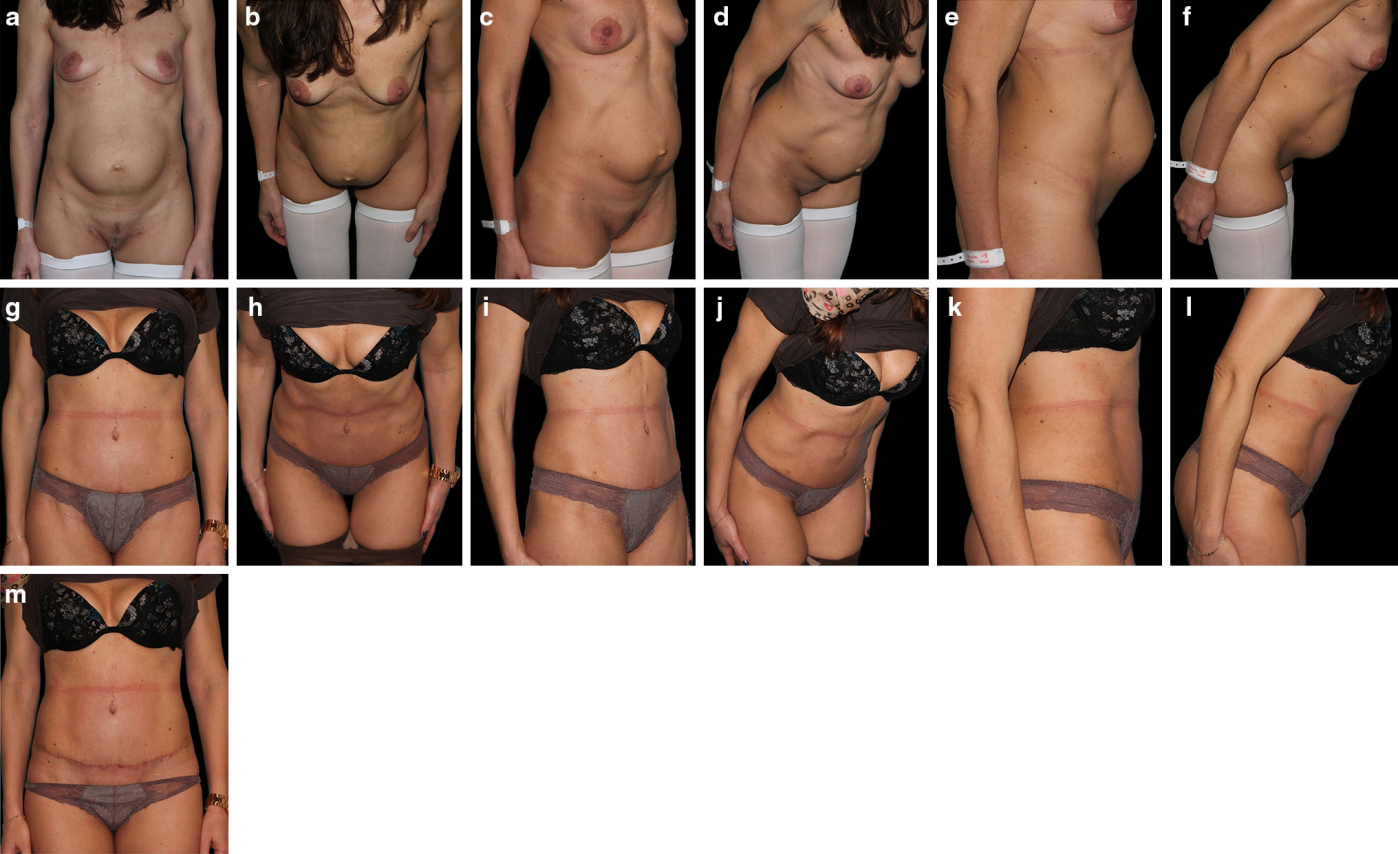
**a-f** representative patient before surgery. **g-m** representative patient after surgery

46-year-old patient, BMI 29, 2 cesarean surgeries, 9cm RD with a concomitant umbilical hernia (treated with nylon 0.0 sutures-no mesh). The patient has not maintained any training program. Representative patient examples before and after surgery are shown in Fig. 4a–m.

### Case 2

**Fig. 5:**
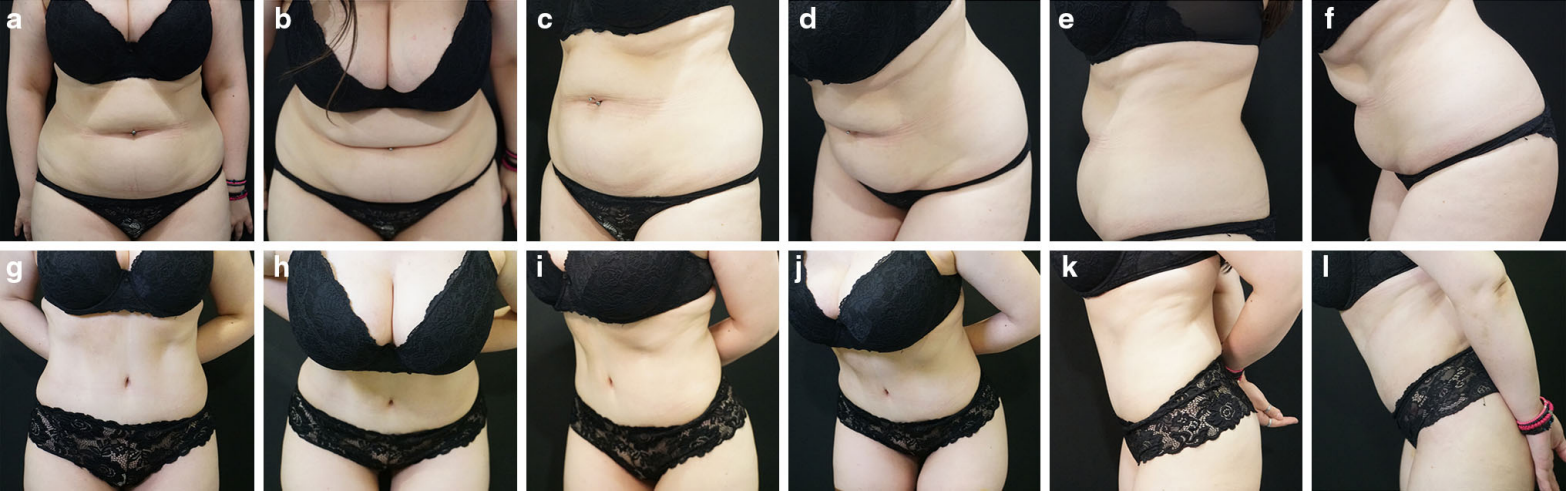
**a-f** representative patient before surgery. **g-l** representative patient after surgery

32-year-old patient, BMI 31, 4cm RD. The patient has not maintained any training program. Representative patient examples before and after surgery are shown in Fig. 5a–l.

## Discussion

This study provides a classification, treatment, and evaluation algorithm for the management of RD in abdominoplasties that every plastic surgeon can easily adapt. The zero recurrence rate in 124 patients treated for RD up to 9cm using absorbable sutures and low postoperative complications and pain establish this treatment protocol as a safe and adequate tool for RD correction.

Previous classifications include the Nahas [7, 17] classification based on the myofascial deformity, the Rath [7] classification based on the attenuation level relative to the umbilicus and the patient age, and the Beer [9] classification based on the normal width of the linea alba. Recently, in 2019, the International endohernia society (IES) [3] proposed a classification of Rectus diastasis (RD) with concomitant hernias based on its location (subxiphoidal, epigastric, umbilical, infraumbilical, suprapubic) and width (mild < 3 cm, moderate = 3−5 cm, and severe> 5 cm). Our study's classification has been investigated since 2014, and they have a lot of similarities. The main difference is that an RD of more than 5cm is further categorized as severe (5−7cm) and very severe (7−9cm). The reason is that a higher tension that will exist during this plication could pose a greater risk of long-term recurrence. Moreover, it brings a more precise evaluation to the RD. In the literature, RD more than 6cm in most cases is corrected using reinforced prosthetic meshes to avoid possible recurrences [4, 18, 18–20]. These specifications allow the plastic surgeon to differentiate the treatment and evaluate the efficacy of a suture-only technique. Finally, while it has been described that abdominal wall protrusion is the main indication for surgery, we find this parameter useful but difficult to measure objectively and use as treatment guidance [21]

Type A (2−3cm) is considered mild diastasis, and often patients are not aware of its existence preoperatively. Clinically it is diagnosed using the finger-width method. For its correction, only one suture layer is used to correct this mild diastasis. Gama et al. [22] demonstrated a zero recurrence rate when used in patients (Group I) with an intraoperative inter-rectus distance up to 2.8cm. Type B (3-5cm) is considered moderate diastasis, and patients usually complain about a bulge when they contract their abdominal muscles. Clinically it is best observed while the patient tries to sit up from a supine position, causing the abdominal muscles to contract and make the diastasis more evident. A two-layer suture is used in this case to correct the diastasis. It has also been reported in the literature by Rodrigues et al. [23] and Nahas et al. It has been used in the intraoperative measured inter-rectus distance up to 4.5cm and has been proven to be an adequate treatment method with zero recurrence rate [24–28].different techniques have been described to treat severe RD; however, there is a lack of consensus concerning the best treatment. Type C (5−7cm) is considered severe diastasis, and patients usually present with a protruding midline or an abdominal bulge in a standing position even when they do not contract their abdominal muscles. A two-layer suture is used as well in this category, but more interrupted sutures are placed along the RD (1cm distance between them versus 2cm distance in type B). This way, a tension-free suture is established, recurrence is avoided, and aesthetically pleasing midline contour is achieved. Type D (7−9cm) is considered very severe RD, and patients present with obviously distorted rectus muscles anatomy, prominent abdominal bulge both in sitting and standing position and are severely dissatisfied with their appearance. As reported in the literature in diastasis, more than 6−7cm mesh placement is the only available treatment approach to our knowledge. Our goal was to provide our patients with a mesh-free abdominoplasty because of the additional potential risks and increased cost. For that reason, we developed the ouroboros technique as mentioned above. This maneuver creates three suture layers that manages to re-establish correct rectus muscles anatomy, decreases the rectus sheath tension, and does not create further muscle damage during suturing.

This study evaluates RD clinical recurrence, complications rate, and quality of life characteristics between the four RD types. The recurrence rate was zero and was assessed at least 24 months postoperatively using the finger-width method. It has been reported that it is a validated method in recognizing clinical RD recurrences [10, 11]. Moreover, it is a safe, patient-friendly, and inexpensive evaluation method contrary to other methods such as U/S, MRI, or CT. In addition, recurrence of rectus diastasis diagnosed by ultrasonography is not always related to a clinically identifiable deformity [29]. Focusing on clinical and not on imaging recurrence of RD became a central figure in the postoperative evaluation. Also, preoperative consultation in a comfortable setting along with the surgeon to help the patient become aware of the existing condition and understand the final aims of the surgery is of significant importance and contributes to the high postoperative satisfaction rate.

Previous studies had different recurrence rates ranging from 0 up to 100% [27, 30–32]. This significant deviation has been attributed to multiple reasons such as the type of sutures, number of layers, and direction of RD plication. It is all already demonstrated that absorbable and non-absorbable sutures are equally effective without differences in recurrence rate [19, 27, 33]. In this study, absorbable sutures are used due to the lower risk of inflammatory reactions/granuloma formation and suture palpation in thinner patients. In the aforementioned group of patients, interrupted sutures could be placed before CIS sutures to avoid any palpations issues until suture absorption is completed. Regarding the number of layers, the main considerations are surgical time and increase of abdominal pressure. While the recurrence rate is the same, the complication rate is lower in the double layer [4]. While a double layer has been reported to require 20minutes longer [22] in our studies, surgical time for a triple-layer used in Type D was 18minutes. There was no correlation between surgical time and complications rate, even in combination surgeries that ranged between 2 and 6 hours. Thus a double or triple-layer suture is not posing any risk to the overall patient's health. An alternative in these cases, as found in the literature, is the use of a mesh that requires significant time and is linked to an increased risk of infection, even chronic pain due to nerve damage from fibrosis generated from mesh absorption. Thus, more evidence is needed to consider it as a first-line treatment [34–36].

Complications rates were similar to the ones found in the literature [4]. During patient selection, in contrast to most studies, smokers are not excluded, and the main reason is that Greece still has one of the world's highest smoking rates [37]. In this study smoking population was 44.35%. Since smoking has been linked to a higher rate of complications, we advised our patients to eliminate smoking four weeks preoperatively and until primary healing, two weeks postoperatively [38]. However, in this population, statistical analysis did not show any correlation to smoking and complications rate.

Regarding Intra-abdominal pressure (IAP), it is essential to avoid wide plications while approaching the muscles. The reason for that is the possible increase of the IAP. The use of compressive garments can also aggravate IAP [39]. However, these changes are transient and most times resolve within the fifth postoperative day [40]. For that reason, in patients with RD Types C and D who underwent liposuction to the flanks, compressive garments are avoided the first 10−15 postoperative days. The main reason we use compressive garments is not to prevent seroma but to avoid edema caused by liposuction of the flanks. However, as the study evolved, we stopped using compressive garments to prevent a possible increase of the IAP. Plications may also impair pulmonary function by decreasing pulmonary compliance [41]. Spirometric parameters have also been shown to normalize the 15th day [39]. Previous studies suggest that these changes are transient and do not pose any potential clinical risk to healthy individuals. However, precaution measures that lower the risk of DVT and pneumonic embolism such as Flowtron boots, bent knees at 10−15° during surgery, electric blanket, and surgical room temperature set at 20−22°C to avoid hypothermia and early mobilization are of great importance [42].

Also, patients' quality of life characteristics was evaluated through postoperative questionnaires. Overall pain score was low, and no statistically significant differences were observed between the four RD types. All patients received perioperatively ropivacaine [43] injections into the rectus muscle. As indicated from the low pain score levels, RD correction is not associated with increased postoperative pain in abdominoplasty procedures when treated with ropivacaine injections. Thus, extra, time-consuming precautions to alleviate pain, such as nerve blocks, could be avoided [44, 45]. Adequate pain control resulted in early mobilization (within the first 4−6 postoperative hours) and short-term hospitalization (less than 24 hours). Also, in less than a week, most patients had returned to indoor/outdoor activities while no patient-reported work impairment. Forty-two patients started and maintained a typical exercise program after surgery, and none of them experienced discomfort during exercise. The overall low pain score and quick return to full function indicate that RD correction is not accompanied by decreased long-term functionality. The evaluation of the aesthetic result using a GAIS 5-point scale revealed a 95.97% satisfaction rate. The reasons that 4.03% of patients were not fully satisfied were scar complications, and all of them were re-operated to address their issues.

One limitation of this study is that the patient quality of life assessment did not include a validated instrument such as the BODY-Q [46] used in body contouring surgeries. The reason for that is that the study design started in 2014, and thus, there was a lack of validated questionnaires regarding body contouring surgeries. Also, our study does not include a control group without RD to compare complications, pain, and quality of life characteristics between RD and non RD patients. Another limitation is that our technique has not been examined in RD more than 9cm because such a case did not occur in our patient population. However, if a patient with over 9cm diastasis underwent abdominoplasty, we suggest treating it with ouroboros sutures but with a 1cm distance between the interrupted sutures instead of 2cm. Also, in our study mean BMI was 23.84, with only three patients with a BMI of 31-35. This parameter is essential as increased intra-abdominal fat in obese patients could complicate the approximation of rectus muscles.

This prospective study is one of the few that includes patient satisfaction and patient-reported outcomes. Moreover, to our knowledge, no prospective study or clinical trial has reported outcomes using only sutures to repair diastasis over 6cm [4, 19, 20]. While there is still no consensus concerning the optimal way to correct RD, our proposal aims to provide surgeons a safe treatment guide. This is the first study to support that a simple suture-based technique that almost every plastic surgeon can use is enough to correct RD. Since RD has been proven that it is not a true hernia, the use of sutures is more than adequate for its correction, and there is no need for extreme measures.

It is our hope that our study will be a valuable tool for the plastic surgery society and could help future surgeons as a treatment guide during abdominoplasty procedures and as a guide on how to report their outcomes in a practical yet viable in a private practice setting manner.

## Conclusions

Several classification systems have been reported to define the spectrum of rectus diastasis. This article presents an updated classification system and treatment protocol to provide surgeons with a safe and standardized method that produces high-quality aesthetic results.

## Supplementary Information

Below is the link to the electronic supplementary material.

Supplementary file1 (mp4 213004 KB)
